# Ictal cold shiver caused by autoimmune limbic encephalitis: A case report and literature review

**DOI:** 10.1016/j.ebr.2025.100773

**Published:** 2025-04-22

**Authors:** Yosuke Takeuchi, Teruaki Masuda, Hiroyuki Matsuta, Kenichi Yabuuchi, Nobuhiro Hata, Minoru Fujiki, Konen Obayashi, Etsuro Matsubara, Noriyuki Kimura

**Affiliations:** a Department of Neurology, Faculty of Medicine, Oita University, Oita, Japan; b Department of Neurosurgery, Faculty of Medicine, Oita University, Oita, Japan; c Department of Clinical Physiology, Graduate School of Life Sciences, Kumamoto University, Kumamoto, Japan

**Keywords:** Cold shiver, Temporal lobe, Autoimmune, Limbic encephalitis, LGI1 antibodies

## Abstract

Ictal cold shiver is a rare manifestation of seizures that originate in the temporal lobes; it is characterized by body trembling and sensations of cold. This study aims to investigate the clinical manifestations and etiological factors in patients with ictal cold shiver. We reviewed 59 cases of ictal cold shiver by using various keywords to search published original articles and case reports, and we described a new case of autoimmune limbic encephalitis. The median age at diagnosis was higher in patients with autoimmune encephalitis than in those with other etiologies (p = 0.007). Autoimmune encephalitis showed more frequent bilateral temporal lobe abnormalities on magnetic resonance imaging (MRI) (p = 0.027) and generalized or bilateral independent epileptiform discharges on electroencephalography (EEG) (p = 0.047). Anti-leucine-rich glioma-inactivated 1 antibodies were most commonly detected in patients with autoimmune encephalitis with ictal cold shiver (85.7 %). This study highlights the importance of considering autoimmune mechanisms in the presence of ictal cold shiver, especially in patients with bilateral temporal lobe lesions on MRI, generalized or bilateral independent epileptiform discharges on EEG, and older onset.

## Highlights

• Ictal cold shiver is a rare seizure manifestation that originates in temporal lobes.
• Autoimmune encephalitis is one cause of ictal cold shiver.
• Anti-LGI1 antibodies may be the most common autoantibodies in this manifestation.

## 1 Introduction

Ictal cold shiver, which is a rare manifestation of seizures that originate in the temporal lobes, is characterized by body trembling accompanied by sensations of cold and may occur concomitantly with goose bumps or other autonomic symptoms [[1], [2], [3]]. Because convulsions and impaired consciousness are often absent, epileptic seizures are not often diagnosed. Although the precise mechanism of this phenomenon remains unclear, the amygdala has been implicated in the etiology of this manifestation. In addition, structural or functional abnormalities of the temporal lobes are hypothesized to contribute to this manifestation, as it is categorized as a seizure symptom of temporal lobe epilepsy [4].

The importance of autoantibodies in seizure management has recently increased, and the International League Against Epilepsy Autoimmune and Inflammation Working Group divided the autoimmune mechanisms into two groups: acute symptomatic seizures secondary to autoimmune encephalitis and autoimmune-associated epilepsy [5]. Acute symptomatic seizures secondary to autoimmune encephalitis occur during the active phase of autoimmune encephalitis and are resistant to conventional antiepileptic pharmacotherapy, so immunomodulatory intervention is necessary [6]. In addition, identification of autoantibodies plays a crucial role in facilitating early diagnosis and treatment in cases of autoimmune encephalitis [7,8].

In this paper, we present a review of the literature on ictal cold shiver and a new illustrative case of autoimmune limbic encephalitis to clarify the clinical features of patients with ictal cold shiver.

## 2 Methods

### 2.1 Literature review

**Fig. 1:**
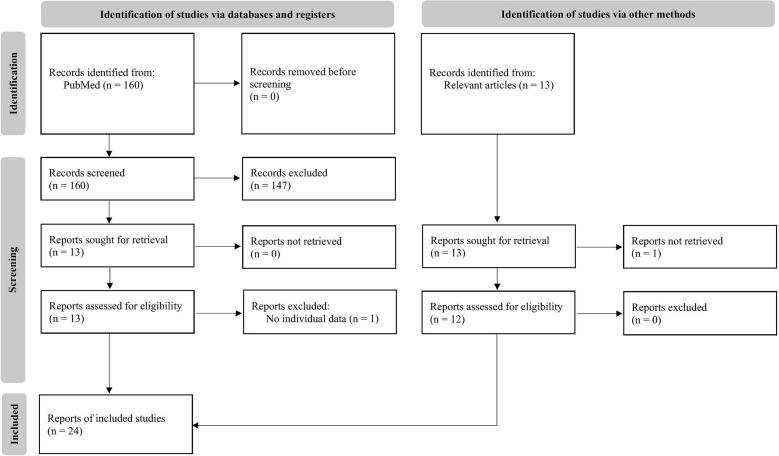
The flowchart of the selection of the articles.

We conducted a detailed systematic review of published original articles and case reports of ictal cold shiver. We used the Preferred Reporting Items for Systematic Reviews and Meta Analyses criteria for the systematic review [9]. We searched PubMed with a combination of the keywords “pilomotor” or “piloerection” or “piloerections” or “goose bump” or”goose bumps” or “shiver” or “shivers” or “chill” or “chills” or “shudder” or “shudders” and “ictal” or “epilepsy” or “seizure” or “encephalitis” for studies published until August 24, 2024. We also manually searched the reference lists of relevant articles. The articles included reports of patients with cold shiver who had diagnoses of seizure symptoms that were based on clinical signs, electroencephalography (EEG), and magnetic resonance imaging (MRI). Patients were excluded if details of their clinical background, such as age, sex, and seizures, were not available (Fig. 1). We also described the types of autoantibodies in autoimmune encephalitis. We performed statistical analyses by using Student's *t*-test and Fisher’s exact test with JMP 14.0 (SAS Institute Japan, Tokyo, Japan). We set p values of less than 0.05 as statistically significant. This study was approved by the Oita University Research Ethics Committee (No. 2373-D2 and No. 2640). The patient provided written informed consent for participation in the study.

## 3 Results

### 3.1 General characteristics of the patients

Our literature review identified reports of 59 patients with ictal cold shiver [1,2,[10], [11], [12], [13], [14], [15], [16], [17], [18], [19], [20], [21], [22], [23], [24], [25], [26], [27], [28], [29], [30], [31]] (supplemental Table 1, Table 2). Fig. 1 shows a flowchart of the article selection. We analyzed the demographic and clinical characteristics of these patients and the new case (Table 1). The causes of ictal cold shiver were autoimmune encephalitis in 14 patients (23.3 %) and non-autoimmune encephalitis in 46 patients (76.7 %); these latter disorders included cryptogenic neurologic diseases (40.0 %), hippocampal sclerosis (11.7 %), brain tumors (10.0 %), trauma (5.0 %), arteriovenous malformation (3.3 %), congenital diseases (3.3 %), lobectomy (1.7 %), and aneurysm (1.7 %) (supplemental Table 1). The median age at diagnosis in patients with ictal cold shiver with autoimmune encephalitis was higher than that in patients with non-autoimmune encephalitis (59 vs. 40 years, p = 0.007). Although most patients with ictal cold shiver manifested autonomic symptoms (such as goose bumps, nausea, flushing, palpitation, sweat, and pallor), five patients (8.3 %) experienced cold shiver independently. The most common seizure classification was focal awareness seizures (FAS), which were present in 90 % of patients; focal motor seizures occurred in 16.7 % of patients; and focal to bilateral tonic-clonic seizures occurred in 15 % of patients. MRI findings were described in 36 patients, 31 (86.1 %) of whom had abnormal intensities and/or atrophy in the temporal lobes. Patients with autoimmune encephalitis had more frequent abnormal findings in the bilateral temporal lobes than did those with non-autoimmune encephalitis (30.0 % vs. 0 %, p = 0.027). Among the 40 patients who had EEG findings, 39 had epileptiform discharges at the time of the seizure and 35 patients had localized epileptiform activities based on the American Clinical Neurophysiology Society’s Standardized Critical Care EEG Terminology: 2021 Version [32]. Patients with autoimmune encephalitis had more generalized or bilateral independent epileptiform discharges than did those with non-autoimmune encephalitis (25.0 % vs. 0 %, p = 0.047).

Table 2 provides the clinical characteristics of autoimmune encephalitis with ictal cold shiver in 14 patients, as detailed in the literature. Anti-leucine-rich glioma-inactivated 1 (LGI1) antibodies were detected in 12 patients (85.7 %), followed by anti-voltage-gated potassium channel antibodies and anti-contactin-associated protein 2 antibodies. The most common ictal symptom with cold shiver was goose bumps, which were present in 71.4 % of patients. Three (21.4 %) patients had faciobrachial dystonic seizures (FBDS). Two patients manifested cold shiver as the only ictal symptom and tested positive for anti-LGI1antibodies. Brain MRI studies showed abnormal intensities in the temporal lobes in 10 patients. Ictal EEG findings revealed epileptiform discharges in all patients except for four patients for whom no findings were described.

**Table 1:** Demographics and clinical characteristics of study subjects.

Measure	Total population (n = 60)	Patients with autoimmune encephalitis (n = 14)	Patients with non-autoimmune encephalitis (n = 46)	p value
Age, years, median (range)	42 (22–76)	59 (35–71)	40 (22–76)	0.007
Sex, male, n (%)	29/60 (48.3)	8/14 (57.1)	21/46 (45.7)	0.547
				
Cold shiver, n (%)				
Autonomic symptoms with cold shiver	50/60 (83.3)	10/14 (71.4)	40/46 (87.0)	0.222
Cold shiver only	5/60 (8.3)	2/14 (14.3)	3/46 (6.5)	0.582
				
Seizure classification, n (%)				
FAS	54/60 (90.0)	11/14 (78.6)	43/46 (93.5 %)	0.133
FMS	10/60 (16.7)	4/14 (28.6)	6/46 (13.0)	0.222
FBTCS	9/60 (15.0)	4/14 (28.6)	5/46 (10.9)	0.193
				
Brain MRI, n (%)				
Temporal lobe abnormalities	31/36 (86.1)	10/12 (83.3)	21/24 (87.5)	1.000
Bilateral lesions	3/31 (9.7)	3/10 (30.0)	0/21 (0)	0.027
Left lesions	16/31 (51.6)	3/10 (30.0)	13/21 (61.9)	0.135
				
Localization of epileptiform activities on EEG, n (%)				
Lateralized	31/35 (88.6)	4/8 (50.0)	27/27 (100.0)	0.001
Generalized	2/35 (5.7)	2/8 (25.0)	0/27 (0)	0.047
Bilateral independent	2/35 (5.7)	2/8 (25.0)	0/27 (0)	0.047

FAS: focal awareness seizures, FMS: focal motor seizures, FBTCS: focal to bilateral tonic-clonic seizures, MRI: magnetic resonance imaging, EEG: electroencephalography.

**Table 2:** Literature review of 14 cases of autoimmune encephalitis with ictal cold shiver.

Case number	Authors, year, reference	Age/sex	Antibodies	Ictal symptoms other than cold shiver	Seizure classification	Imaging findings (CT or MRI)	Ictal EEG findings
1	Wieser et al., 2005	42/M	VGKC	Goose bumps, flushing, fear, olfactory illusion, disorientation, amnesia	FAS, FNMS	Abnormal intensities in the bilateral hippocampi	Theta activities in bilateral temporal lobes
2	Quek et al., 2012	71/M	LGI1	Goose bumps, euphoria, laughter, nonsensical speech, bilateral upper extremity jerking, confusion, generalized tonic-clonic seizures	FIAS, FMS, FNMS, FBTCS	Abnormal intensity in the left medial temporal lobe	Slow waves in left temporal lobe
3	Baysal-Kirac et al., 2016	35/M	CASPR2	Goose bumps, epigastric symptom, secondary generalized seizure	FAS, FNMS, FBTCS	Left hippocampal sclerosis	Seizure from left frontal and temporal lobes, nonlocalized seizure
4	Aurangzeb et al., 2017	64/F	LGI1	Goose bumps, vocalizations, automatisms, FBDS, unresponsive	FIAS, FMS, FNMS	ND	Epileptiform discharges in right frontal and left temporal lobes
5		53/M	LGI1	FBDS	FAS, FMS, FNMS	ND	ND
6	Finke et al., 2017	48/M	LGI1	Goose bumps, complex focal seizures with secondary generalization	FIAS, FNMS, FBTCS	Abnormal intensity in right temporal lobe	Rhythmic theta activities in bilateral temporal lobes
7		68/F	LGI1	FBDS	FAS, FMS, FNMS	Abnormal intensities in bilateral hippocampi	ND
8		60/M	LGI1	None	FAS, FNMS	Abnormal intensity in right hippocampus	Generalized slowing
9	Wennberg, et al., 2018	35/F	LGI1	Goose bumps, diaphoresis, pallor, generalized tonic‐clonic seizure	FAS, FNMS, FBTCS	Bilateral abnormal intensities in mesial temporal lobes	Rhythmic 2–5 Hz activities in bilateral temporal areas
10	Lindgren, et al., 2020	68/M	LGI1	Goose bumps, fear, anxiety, palpitation	FAS, FNMS	High-signaling changes in right temporal lobe	Seizure activity in right temporal area
11	Sun et al., 2023	58/M	LGI1	Goose bumps	FAS, FNMS	Normal	ND
12		40/F	LGI1	Goose bumps	FAS, FNMS	Normal	ND
13		61/F	LGI1	Goose bumps	FAS, FNMS	Abnormal signal and swelling of right hippocampus	Temporal ictal activity
14	New case, 2024	66/F	LGI1	None	FAS, FNMS	Abnormal intensity in left medial temporal lobe	Epileptiform discharges in left temporal lobe

CT: computed tomography, MRI: magnetic resonance imaging, EEG: electroencephalography, M: male, F: female, VGKC: anti-voltage-gated potassium channel antibody, LGI1: anti-leucine-rich glioma-inactivated 1 antibody, CASPR2: anti-contactin-associated protein 2 antibody, FBDS: faciobrachial dystonic seizure, FAS: focal awareness seizure, FNMS: focal nonmotor seizure, FIAS: focal impaired awareness seizure, FMS: focal motor seizure, FBTCS: focal to bilateral tonic-clonic seizure, ND: not described.

### 3.2 New illustrative case

**Fig. 2:**
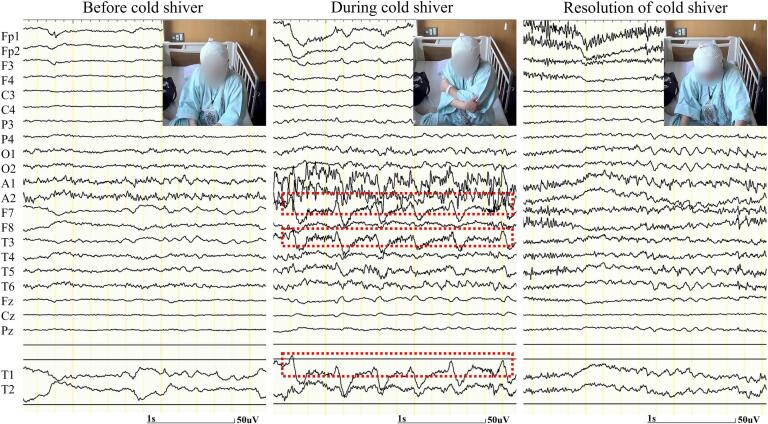
Video-electroencephalographic monitoring. The electroencephalogram (bipolar montage; high-cut filter, 60 Hz; time constant, 0.3 s) showed the lateralized periodic discharges at F7, T1 (left anterior-temporal), and T3 (left mid-temporal), which were time-locked to cold shiver (dotted line areas).

**Fig. 3:**
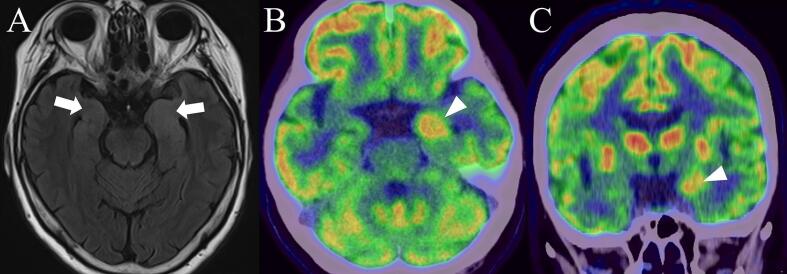
Brain magnetic resonance imaging and [^18^F]fluorodeoxyglucose ([^18^F]FDG) positron emission tomography (PET). (A) Fluid-attenuated inversion recovery imaging showed high intensity and enlargement of bilateral amygdalae with left dominance (arrows). (B and C) [^18^F]FDG PET revealed increased uptake in the left amygdala (arrowheads).

A 66-year-old woman was admitted to our hospital with a 6-month history of paroxysmal shivering with feeling cold even though the ambient temperature was not low; the shivering lasted for 1–2 min and occurred 6 or 7 times per day. This involuntary movement was characterized by rhythmically synchronized trembling of the extremities and trunk, which was distinct from myoclonus or dystonic postures. During the shivering episode, she had no other symptoms, including goose bumps, emotional symptoms, automatism, or impaired awareness. The Mini-Mental State Examination (MMSE) score and working memory index of the Wechsler Adult Intelligence Scale-IV (WAIS-IV) were 24 and 74 points, respectively. Video EEG monitoring showed lateralized periodic discharges in the left temporal region, which were time-locked to shivering without evolving or fluctuating (Fig. 2). Brain MRI revealed abnormal intensity and enlargement of the bilateral amygdalae with left dominance (Fig. 3A). Brain [^18^F]fluorodeoxyglucose ([^18^F]FDG) positron emission tomography (PET) demonstrated increased uptake in the left amygdala (Fig. 3B and C). Cerebrospinal fluid analysis revealed no pleocytosis, normal protein levels, and no oligoclonal bands. Serum anti-LGI1 antibody test results were positive. Additional analyses showed normal anti-myelin oligodendrocyte glycoprotein, paraneoplastic autoantibodies, and anti-*N*-methyl-d-aspartate receptor antibodies. These findings led us to believe that ictal cold shiver was an electroclinical seizure associated with temporal lobe lesions induced by anti-LGI1 limbic encephalitis. Antiepileptic drugs did not effectively relieve symptoms, but the administration of steroid pulse therapy (methylprednisolone, 1000 mg/day for 3 days) followed by oral prednisolone (25 mg/day) reduced the seizure frequency. In addition, we initiated intravenous immunoglobulin therapy, which led to the complete resolution of the ictal cold shiver. On the basis of the clinical course, a diagnosis of acute symptomatic seizures secondary to anti-LGI1 limbic encephalitis was determined. At the time of discharge, the head MRI continued to show amygdala enlargement, whereas the MMSE score and working memory index of the WAIS-IV improved to 28 and 76 points, respectively.

## 4 Discussion

In this paper, we reviewed the literature on cases of ictal cold shiver and presented a new illustrative case of anti-LGI1 limbic encephalitis with ictal cold shiver. Several studies reported cases of ictal goose bumps, or piloerection. However, to our knowledge, this report is the first to fully describe the clinical features of ictal cold shiver. In addition, we found that autoimmune encephalitis may be an important cause of ictal cold shiver.

Ictal cold shiver is a rare manifestation of focal seizures often accompanies goose bumps, which are classified as autonomic symptoms [[1], [2], [3]]. In our review, 83.3 % of the cases with ictal cold shiver presented with autonomic symptoms, including goose bumps, nausea, flushing, palpitation, sweat, and pallor. Although the seizure classification of patients with ictal cold shiver is mostly FAS, which does not involve impaired consciousness or convulsions, diagnosing epileptic seizure from this symptom alone is often difficult [33]. In addition, five patients (5/60) developed ictal cold shiver without other manifestations [1,2,14]. These findings suggest that early diagnosis and treatment of seizures may be delayed if epileptic seizures are not suspected in ictal cold shiver.

Our review indicates that ictal cold shiver originates from the temporal lobes, especially the left [1,2]. How does ictal cold shiver develop in patients with temporal lobe lesions? One explanation is that the amygdala may be the origin of the cold shiver. Cold shiver is a thermogenic response to cold sensations in humans [[34], [35], [36]]. A previous report suggested that the lateral parabrachial nucleus, a mediator of cutaneous thermosensory signals, may project to the central amygdala nucleus by means of cold exposure, which would lead to cold avoidance behaviors including cold shiver [37]. Therefore, epileptic discharges that spread to the amygdala may affect thermoregulatory function and cause cold shiver in patients with temporal lobe lesions [37]. However, why ictal cold shiver occurs predominantly with left temporal lobe lesions is unclear [1].

We found in our literature review that the most common etiology of ictal cold shiver was cryptogenic (40.0 %) and the second most common was autoimmune encephalitis (23.3 %). However, the extent to which autoantibodies have been investigated in idiopathic cases remains unclear. Moreover, screening for autoantibodies was not as widespread in the past as it is today. Therefore, ictal cold shiver with autoimmune encephalitis may be more common than previously believed. The autoantibodies that were detected in patients with autoimmune encephalitis presenting with ictal cold shiver were anti-LGI1 and related antibodies. FBDS, which is believed to be a specific manifestation of anti-LGI1 encephalitis, was observed in only three cases [14,38]. However, autoimmune encephalitis, which is characterized by ictal piloerection, an autonomic seizure manifestation, is frequently associated with anti-LGI1 antibodies [17]. Therefore, ictal cold shiver may serve as a significant indicator for the diagnosis of anti-LGI1 encephalitis. Additional research is necessary to clarify the relationship between ictal cold shiver and anti-LGI1 encephalitis.

Our findings thus demonstrated the distinction between ictal cold shiver of autoimmune encephalitis and ictal cold shiver of other etiologies. Patients with ictal cold shiver of autoimmune encephalitis were older than patients with ictal cold shiver of other etiologies. Anti-LGI encephalitis, which constituted the majority of autoimmune etiologies, has reportedly developed at a mean age of 60 years [39], which may explain the observed trend toward the older age at onset in the cohort of patients with ictal cold shiver of autoimmune encephalitis. However, we observed no differences in seizure classification between the two groups, which indicates a need for a distinguishing factor in clinical practice. MRI findings in the autoimmune encephalitis group revealed bilateral lesions or atrophy of the temporal lobes at a frequency of 30 %, whereas the group with other etiologies had no such findings. When physicians encounter cases of ictal cold shiver with delayed onset or bilateral atypical lesions on MRI scans, investigating the possibility of an autoimmune pathology is recommended [40]. With regard to EEG findings, cases with detailed descriptions predominantly manifested ictal epileptiform discharges. Notably, the frequency of generalized or bilateral independent epileptic findings was higher in patients with autoimmune encephalitis than in patients with other etiologies. Our review indicated that the EEG finding of generalized or bilateral independent epileptiform discharges may be a characteristic feature of autoimmune encephalitis in cases with ictal cold shiver. Several studies also suggested that generalized seizures are clinical features of autoimmune encephalitis [41,42], and careful attention should be paid to EEG findings in addition to results of imaging investigations in the diagnosis of autoimmune encephalitis. Delayed recognition and treatment of autoimmune encephalitis frequently lead to severe residual deficits [43]. Therefore, clinical evaluation of potential autoimmune mechanisms is of paramount importance in the management of these conditions.

Our study has certain limitations. First, we carefully searched the literature, but our review did not include previous reports that could not be extracted from PubMed. Second, our case and previous studies were limited to small cohorts and case reports, and the definitions of imaging and EEG abnormalities were not standardized. Finally, autoantibodies were analyzed by using various detection techniques, and the types of autoantibodies to be analyzed were not standardized. Therefore, a multicenter prospective study with a large sample size is needed to address these issues.

## 5 Conclusion

In this study, we identified ictal cold shiver as a potential manifestation of autoimmune limbic encephalitis that occurred with greater frequency than anticipated, particularly in anti-LGI1-related encephalitis. In the management of patients experiencing ictal cold shiver, being alert to MRI and EEG abnormalities indicative of autoimmune encephalitis is crucial, as is investigating autoantibodies so as to improve the prognosis of patients.

## Ethical Statement

This study was approved by the Oita University Research Ethics Committee (No. 2373-D2 and No. 2640). The patient provided written informed consent for participation in the study.

## CRediT authorship contribution statement

**Yosuke Takeuchi:** Writing – review & editing, Writing – original draft, Visualization, Resources, Methodology, Investigation, Formal analysis, Conceptualization. **Teruaki Masuda:** Writing – review & editing, Writing – original draft, Visualization, Supervision, Project administration, Methodology, Investigation, Funding acquisition, Conceptualization. **Hiroyuki Matsuta:** Writing – review & editing, Resources, Investigation. **Kenichi Yabuuchi:** Writing – review & editing, Investigation, Formal analysis. **Nobuhiro Hata:** Writing – review & editing, Resources, Investigation. **Minoru Fujiki:** Writing – review & editing, Resources, Investigation. **Konen Obayashi:** Writing – review & editing, Formal analysis. **Etsuro Matsubara:** Writing – review & editing, Supervision. **Noriyuki Kimura:** Writing – review & editing, Supervision.

## Funding

This research was supported by Grant-in-Aid for Scientific Research (C) Grant number 23K06911.

## Declaration of competing interest

The authors declare that they have no known competing financial interests or personal relationships that could have appeared to influence the work reported in this paper.
