# Supplementary material for: Ictal cold shiver caused by autoimmune limbic encephalitis: A case report and literature review
Source: Epilepsy Behav Rep. 2025 Apr 22;30:100773. doi: 10.1016/j.ebr.2025.100773 (PMC12434993; doi:10.1016/j.ebr.2025.100773)
Supplement: Supplementary Data 1 [file mmc1.docx]

**Supplemental Table 1.**

**Clinical characteristics of patients with ictal cold shiver.**

| Authors, year | Age | Sex | Cause | Antibodies | Ictal symptoms other than cold shiver | Seizure classification |
| --- | --- | --- | --- | --- | --- | --- |
| Autoimmune encephalitis |  |  |  |  |  |  |
| Wieser et al., 2005 | 42 | M | AE | VGKC | Goose bumps, flushing, fear, olfactory illusion, disorientation, amnesia | FAS, FNMS |
| Quek et al., 2012 | 71 | M | AE | LGI1 | Goose bumps, euphoria, laughter, nonsensical speech, bilateral upper extremity jerking, confusion, generalized tonic-clonic seizures | FIAS, FMS, FNMS, FBTCS |
| Baysal-Kirac et al., 2016 | 35 | M | AE | CASPR2 | Goose bumps, epigastric symptom, secondary generalized seizure | FAS, FNMS, FBTCS |
| Aurangzeb et al., 2017 | 64 | F | AE | LGI1 | Goose bumps, vocalizations, automatisms, FBDS, unresponsive state | FIAS, FMS, FNMS |
|  | 53 | M | AE | LGI1 | FBDS | FAS, FMS, FNMS |
| Finke et al., 2017 | 48 | M | AE | LGI1 | Goose bumps, complex focal seizures with secondary generalization | FIAS, FNMS, FBTCS |
|  | 68 | F | AE | LGI1 | FBDS | FAS, FMS, FNMS |
|  | 60 | M | AE | LGI1 | None | FAS, FNMS |
| Wennberg et al., 2018 | 35 | F | AE | LGI1 | Goose bumps, diaphoresis, pallor, generalized tonic‐clonic seizure | FAS, FNMS, FBTCS |
| Lindgren et al., 2020 | 68 | M | AE | LGI1 | Goose bumps, fear, anxiety, palpitation | FAS, FNMS |
| Sun et al., 2023 | 58 | M | AE | LGI1 | Goose bumps | FAS, FNMS |
|  | 40 | F | AE | LGI1 | Goose bumps | FAS, FNMS |
|  | 61 | F | AE | LGI1 | Goose bumps | FAS, FNMS |
| New case, 2024 | 66 | F | AE | LGI1 | None | FAS, FNMS |
| Other etiologies |  |  |  |  |  |  |
| Landau et al., 1953 | 22 | M | C | ND | Goose bumps, pale, fading-away of sounds, feeling of strangeness and unreality, sadness, fear, cold sensation, generalized convulsion | FAS, FNMS, FBTCS |
| Mulder et al., 1954 | 25 | F | Congenital | ND | Goose bumps, feeling of quivering in the heart, loss of consciousness | FIAS, FNMS |
| Andermann et al., 1984 | 61 | M | T | ND | Goose bumps, warmth, cold, vibration | FAS, FNMS |
| Green et al., 1984 | 44 | M | T | ND | Goose bumps, jerking movement, epigastric sensation, sense of unpleasant odor | FAS, FMS, FNMS |
| Lesser et al., 1985 | 37 | M | Lobectomy | ND | Goose bumps | FAS, FNMS |
| Yu et al., 1998 | 37 | M | Trauma | ND | Goose bumps, palpitation, fear | FAS, FNMS |
| Roze et al., 2000 | 66 | M | C | ND | Goose bumps, epigastric sensation with a feeling of thoracic oppression, flushing, sensation of hot liquid, disorientation | FAS, FNMS |
| Stefan, Pauli et al., 2002 | 52 | F | C | ND | Nausea, tears | FAS, FNMS |
|  | 46 | M | HS | ND | None | FAS, FNMS |
|  | 37 | F | C | ND | Palpitations, sweats | FAS, FNMS |
|  | 41 | F | T | ND | Nervousness, nausea | FAS, FNMS |
|  | 33 | M | C | ND | Strange feeling of coldness, visual illusion people or things are getting smaller | FAS, FNMS |
|  | 42 | M | HS | ND | Nausea, vertigo | FAS, FNMS |
|  | 41 | F | C | ND | Sweats, flushes, nausea | FAS, FNMS |
|  | 75 | F | C | ND | Current-like feeling, mushroom-like taste, teeth clapping, feeling of heat, nausea | FAS, FMS, FNMS |
|  | 29 | M | Congenital | ND | Goose bumps, grinding feeling in both temples | FAS, FNMS |
|  | 37 | F | C | ND | Palpitations, nausea, vertigo | FAS, FNMS |
|  | 58 | F | AM | ND | Nausea, feeling of pressure in jaw | FAS, FNMS |
| Sa'adah et al., 2002 | 26 | M | Trauma | ND | Palpitation, sweating, epigastric discomfort, fear, loss of consciousness, secondary generalized tonic-clonic seizures | FIAS, FNMS, FBTCS |
| Stefan, Feichtinger et al., 2002 | 75 | F | C | ND | Current-like sensation, mushroom-like taste, teeth clapping, nausea | FAS, FMS, FNMS |
|  | 34 | M | HS | ND | Visual illusion as if everything is getting smaller, secondary generalized tonic-clonic seizures | FAS, FNMS, FBTCS |
|  | 47 | M | HS | ND | Goose bumps, tonic-clonic seizures | FAS, FNMS, FBTCS |
|  | 34 | F | C | ND | Secondary generalized tonic or tonic-clonic seizures | FAS, FNMS, FBTCS |
| Stefan et al., 2003 | 39 | M | C | ND | None | FAS, FNMS |
|  | 34 | M | C | ND | None | FAS, FNMS |
|  | 42 | F | C | ND | Sweating, red face | FAS, FNMS |
|  | 34 | M | T | ND | Nausea | FAS, FNMS |
|  | 43 | M | HS | ND | Nausea | FAS, FNMS |
|  | 53 | F | C | ND | Goose bumps, nausea, fear, depression | FAS, FNMS |
|  | 41 | F | Trauma | ND | Goose bumps, epigastric symptom | FAS, FNMS |
|  | 36 | F | C | ND | Goose bumps | FAS, FNMS |
|  | 59 | F | Aneurysm | ND | Goose bumps, nausea | FAS, FNMS |
|  | 30 | M | C | ND | Goose bumps | FAS, FNMS |
|  | 49 | F | T | ND | Goose bumps, palpitation, sweating | FAS, FNMS |
|  | 28 | F | C | ND | Goose bumps, epigastric symptom | FAS, FNMS |
|  | 47 | M | HS | ND | Goose bumps | FAS, FNMS |
|  | 76 | F | C | ND | Goose bumps, depression | FAS, FNMS |
|  | 33 | M | C | ND | Goose bumps, sweating, olfactory symptom | FAS, FNMS |
|  | 38 | F | C | ND | Goose bumps, vertigo, palpitation, nausea | FAS, FNMS |
|  | 38 | F | C | ND | Goose bumps, nausea | FAS, FNMS |
| Dove et al., 2004 | 26 | F | HS | ND | Goose bumps, fear, panic, feelings of warmth, nausea, lip smacking, altered awareness | FIAS, FMS, FNMS |
| Loddenkemper et al., 2004 | 53 | F | T | ND | Goose bumps, urinary urgency, fear, tachycardia, hyperventilation, face tonic, generalized tonic-clonic seizures | FAS, FMS, FNMS, FBTCS |
|  | 54 | F | AM | ND | Goose bumps, hyperventilation, fear, tachycardia, urinary urgency, automatism | FAS, FMS, FNMS |
| Masnou et al., 2006 | 35 | F | C | ND | Goose bumps, colored phosphenes, nausea, thoracic compression | FAS, FNMS |
| Kurita et al., 2013 | 38 | M | C | ND | Goose bumps, pallor | FAS, FNMS |
| Ando et al., 2024 | 69 | F | C | ND | Sweating | FAS, FNMS |

M: male; F: female; AE: autoimmune encephalitis; C: cryptogenic; HS: hippocampal sclerosis; T: tumor; AM: arteriovenous malformation; VGKC: voltage-gated potassium channel; LGI1: leucine-rich glioma-inactivated 1; CASPR2: contactin-associated protein 2; ND: not described; FBDS: faciobrachial dystonic seizure; FAS: focal awareness seizure; FNMS: focal nonmotor seizure; FIAS: focal impaired awareness seizure; FMS: focal motor seizure; FBTCS: focal to bilateral tonic-clonic seizure.
