# Supplementary material for: Ictal cold shiver caused by autoimmune limbic encephalitis: A case report and literature review
Source: Epilepsy Behav Rep. 2025 Apr 22;30:100773. doi: 10.1016/j.ebr.2025.100773 (PMC12434993; doi:10.1016/j.ebr.2025.100773)
Supplement: Supplementary Data 2 [file mmc2.docx]

**Supplemental Table 2.**

**Imaging and EEG findings in patients with ictal cold shiver.**

| Authors, year | Age | Sex | Imaging findings (CT or MRI) | Interictal EEG findings | Ictal EEG findings |
| --- | --- | --- | --- | --- | --- |
| Autoimmune encephalitis | |  |  |  |  |
| Wieser et al., 2005 | 42 | M | Abnormal intensities in the bilateral hippocampi | Slight slowing of background activity | Ictal theta patterns alternating between the sides, mostly on the left, with contralateral propagation |
| Quek et al., 2012 | 71 | M | Abnormal intensity in left medial temporal lobe | ND | Focal slowing in temporal region, generalized slowing |
| Baysal-Kirac et al., 2016 | 35 | M | Left hippocampal sclerosis | ND | Seizure from left frontal and temporal lobes, nonlocalized seizure |
| Aurangzeb et al., 2017 | 64 | F | ND | ND | Epileptiform discharges in right frontal and left temporal lobes |
|  | 53 | M | ND | ND | ND |
| Finke et al., 2017 | 48 | M | Abnormal intensity in right temporal lobe | ND | Rhythmic theta activities in bilateral temporal lobes |
|  | 68 | F | Abnormal intensities in bilateral hippocampi | ND | ND |
|  | 60 | M | Abnormal intensity in right hippocampus | ND | Generalized slowing |
| Wennberg et al., 2018 | 35 | F | Bilateral abnormal intensities in mesial temporal lobes | Right anterior temporal epileptiform discharges | Rhythmic 2–5 Hz activities in bilateral temporal areas sequentially |
| Lindgren et al., 2020 | 68 | M | High-signaling changes in right temporal lobe | ND | Seizure activity in right temporal area |
| Sun et al., 2023 | 58 | M | Normal | Sharp slow waves in left frontal and anterior middle temporal areas | ND |
|  | 40 | F | Normal | Sharp and slow waves in left anterior middle temporal areas | ND |
|  | 61 | F | Abnormal signal and swelling in right hippocampus | Slow waves in bilateral frontal and anterior middle temporal areas | Temporal ictal activity |
| New case, 2024 | 66 | F | Abnormal intensity in left medial temporal lobe | Normal | Epileptiform discharges in left temporal lobe |
| Other etiologies | |  |  |  |  |
| Landau et al., 1953 | 29 | M | ND | No clearly focal or lateralized process | ND |
| Mulder et al., 1954 | 25 | F | ND | Mild nonfocal abnormality | Sharp waves arising from region of left sylvian fissure |
| Andermann et al., 1984 | 61 | M | Tumefactive lesion in right anterior portion of temporal lobe, insula, basal ganglia | ND | ND |
| Green et al., 1984 | 44 | M | Tumefactive lesion in right temporal lobe | Diffuse slow wave (postictal state) | Rhythmic discharge of sharp and slow waves in right frontal and temporal regions |
| Lesser et al., 1985 | 37 | M | ND | No epileptic activity | Paroxysmal alpha activity in left paroxysmal region |
| Yu et al., 1998 | 33 | M | Subarachnoid hemorrhage | ND | Rhythmic discharges in left temporal lesion |
| Roze et al., 2000 | 66 | M | Normal | ND | Sharp wave discharges at theta frequencies in left frontotemporal region |
| Stefan, Pauli et al., 2002 | 52 | F | Abnormal finding in left temporal lobe | ND | ND |
|  | 46 | M | Abnormal finding in left temporal lobe | ND | ND |
|  | 37 | F | Abnormal finding in left temporal lobe | ND | ND |
|  | 41 | F | Abnormal finding in right temporal lobe | ND | ND |
|  | 33 | M | Abnormal finding in left temporal lobe | ND | ND |
|  | 42 | M | Abnormal finding in right temporal lobe | ND | ND |
|  | 41 | F | Abnormal finding in left temporal lobe | ND | ND |
|  | 75 | F | Normal | ND | Normal |
|  | 29 | M | Abnormal finding in left temporal lobe | ND | ND |
|  | 37 | F | Abnormal finding in left temporal lobe | ND | ND |
|  | 58 | F | Abnormal finding in left temporal lobe | ND | ND |
| Sa'adah et al., 2002 | 26 | M | Atrophy in apex of left temporal lobe | Sharp-wave complexes and slow wave discharges in left temporal lobe | ND |
| Stefan, Feichtinger et al., 2002 | 75 | F | Normal | ND | Rhythmic theta activity in left anterior temporal lobe |
|  | 34 | M | Atrophy in left hippocampus | Spikes in left temporal lobe | Epileptic activity in left temporal lobe |
|  | 47 | M | Abnormal intensity in left temporal lobe | Spikes, sharp waves and theta waves in left temporal lobe | Theta waves in left frontotemporal lobe |
|  | 34 | F | Atrophy in left hippocampus | ND | Epileptic activity in left temporal lobe |
| Stefan et al., 2003 | 39 | M | ND | ND | Epileptic focus in bilateral temporal lobes |
|  | 34 | M | ND | ND | Epileptic focus in left temporal lobe |
|  | 42 | F | ND | ND | Epileptic focus in left temporal lobe |
|  | 34 | M | ND | ND | Epileptic focus in left temporal lobe |
|  | 43 | M | ND | ND | Epileptic focus in left temporal lobe |
|  | 53 | F | ND | ND | Epileptic focus in left temporal lobe |
|  | 41 | F | ND | ND | ND |
|  | 36 | F | ND | ND | ND |
|  | 59 | F | ND | ND | Epileptic focus in left temporal lobe |
|  | 30 | M | ND | ND | Epileptic focus in right temporal lobe |
|  | 49 | F | ND | ND | Epileptic focus in left temporal lobe |
|  | 28 | F | ND | ND | Epileptic focus in right temporal lobe |
|  | 47 | M | ND | ND | Epileptic focus in left temporal lobe |
|  | 76 | F | ND | ND | Epileptic focus in left temporal lobe |
|  | 33 | M | ND | ND | Epileptic focus in left temporal lobe |
|  | 38 | F | ND | ND | Epileptic focus in left temporal lobe |
|  | 38 | F | ND | ND | Epileptic focus in left temporal lobe |
| Dove et al., 2004 | 26 | F | Right mesial temporal sclerosis | ND | Discharges in right anterior temporal lobe |
| Loddenkemper et al., 2004 | 53 | F | Right temporal glioma | ND | Epileptogenic zone in right temporal and temporoparietal areas |
|  | 54 | F | Right superior temporal encephalomalacia after AM rupture | Epileptogenic zone in right temporal and temporoparietal areas | Epileptogenic zone in right temporal area |
| Masnou et al., 2006 | 35 | F | Abnormal intensity of left hippocampus | Normal | Diffuse flattening of electrical activity followed by rhythmic slow activity with maximum amplitude on left central and temporal areas |
| Kurita et al., 2013 | 38 | M | Abnormal intensity in right hippocampus, uncus, amygdala | Normal | Rhythmic wave activity in right temporal lobe |
| Ando, et al., 2024 | 69 | F | Scattered abnormal intensity areas in cerebral white matter, indicative of chronic ischemic changes | Sharp waves and 3-7 Hz slow wave bursts bilaterally in frontal regions | ND |

EEG: electroencephalography; CT: computed tomography; MRI: magnetic resonance imaging; M: male; F: female; ND: not described; AM: arteriovenous malformation.
